# Primary Psoas Abscess Complicated by Septic Arthritis Due to Community-Acquired Methicillin-Resistant Staphylococcus aureus: A Case Report and Review of Literature

**DOI:** 10.7759/cureus.26095

**Published:** 2022-06-19

**Authors:** Abdulrahman M Albouk, Ibrahim M Alhumaidi, Asim J Alamri, Ehab F Alsaygh, Alwaleed A Alshahir, Haneen H Almuhammadi, Rayan S Jamal

**Affiliations:** 1 Department of Orthopedic Surgery, King Fahad Hospital, Medina, SAU; 2 Department of Orthopedic Surgery, Ministry of the National Guard - Health Affairs, Prince Mohammed Bin Abdulaziz Hospital, Medina, SAU; 3 College of Medicine, Taibah University, Medina, SAU; 4 College of Medicine, King Saud Bin Abdulaziz University for Health Sciences, Riyadh, SAU

**Keywords:** cement spacer, total hip replacement (thr), methicillin resistant staphylococcus aureus (mrsa), septic arthritis, psoas abscess

## Abstract

This is a report of an unusual case of a primary psoas abscess due to community-acquired methicillin-resistant *Staphylococcus aureus* (MRSA) in an immunocompetent man. The course of the disease is expressed as septic arthritis of the hip joint with avascular necrosis. Diagnosed one month after symptoms began, the patient was treated by surgical evacuation of the abscess and appropriate antibiotics. Full recovery and return to his usual activity followed total hip replacement.

## Introduction

Iliopsoas muscle abscess (IPA) is the accumulation of pus around the iliopsoas muscle compartment. IPA is a rare entity to cause septic arthritis [1]. A primary abscess usually occurs in the background of diabetes, AIDS, renal failure, intravenous drug abuse, and immunocompromised patients with *Staphylococcus aureus* being the most common causative agent [1,2]. However, our patient was free from co-morbidities, and the IPA was caused by methicillin-resistant *Staphylococcus aureus* (MRSA), which is extremely rare. Therefore, in this report, we describe the case of a primary psoas abscess complicated by septic arthritis due to community-acquired MRSA and shed light on the ambiguous presentation of IPA.

## Case presentation

**Figure 1:**
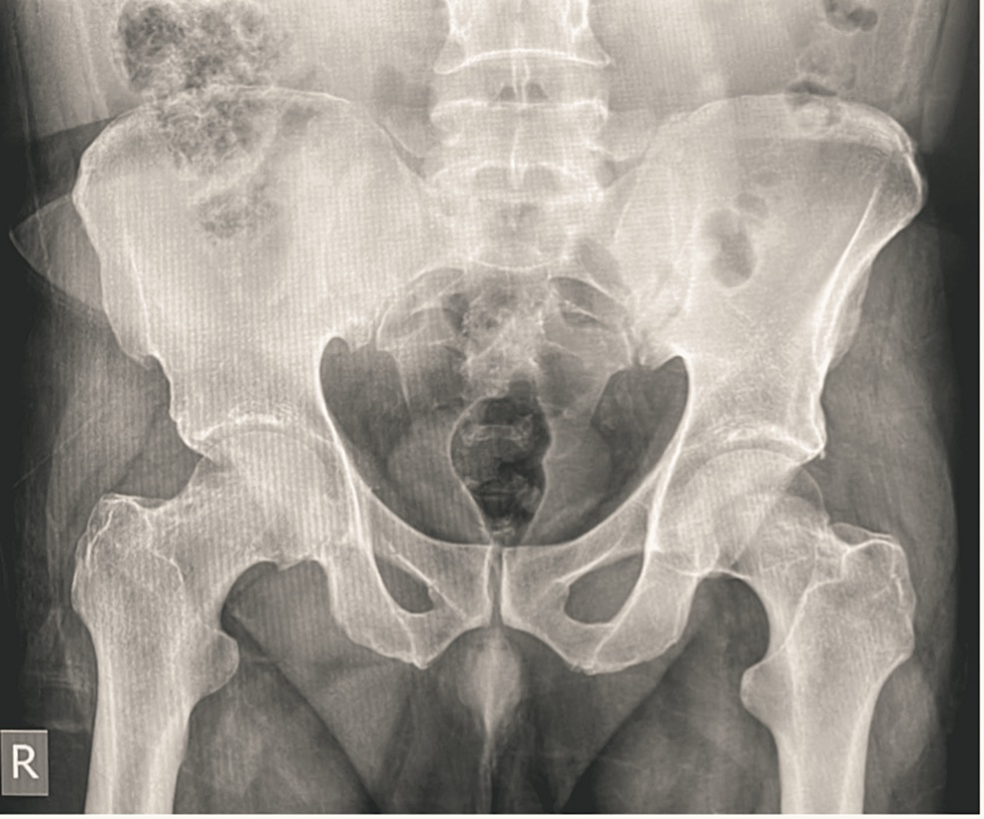
Pelvic x-ray at time of presentation.

A 53-year-old middle-aged gentleman, otherwise free from any co-morbidities, presented multiple times to the emergency department over one month with right hip pain increasing in severity with each visit. Because there was no history of trauma, no constitutional symptoms, and a normal hip radiograph (Figure 1), he was discharged each time with analgesia and muscle relaxants as he was diagnosed with muscle spasms. There were no other radiological or laboratory tests done at the emergency department.

One month after the first visit, the patient returned to the emergency room with severe right hip pain radiating to the right thigh and knee and an inability to bear weight associated with fever, loss of appetite, and weight loss. Upon physical examination, the patient looked unwell, in severe pain, tachycardiac, and febrile (38.9^o^C); however, his Glasgow coma scale (GCS) score was 15/15. Right hip examination showed no redness or warmth but there was severe tenderness over the right hip joint. Right hip range of motion was very limited due to pain and positive iliopsoas sign. Laboratory investigations showed markedly elevated WBC 16.8 x10^9/L, erythrocyte sedimentation rate (ESR) 120 mm/hr, and C-reactive protein (CRP) 420 mg/L. Blood and urine cultures were normal. Tumor markers, brucella titer, QuantiFERON-TB (QIAGEN, Hilden, Germany), and purified protein derivative were negative. A repeated right hip radiograph revealed severe hip osteoarthritis despite being unremarkable one month earlier.

**Figure 2:**
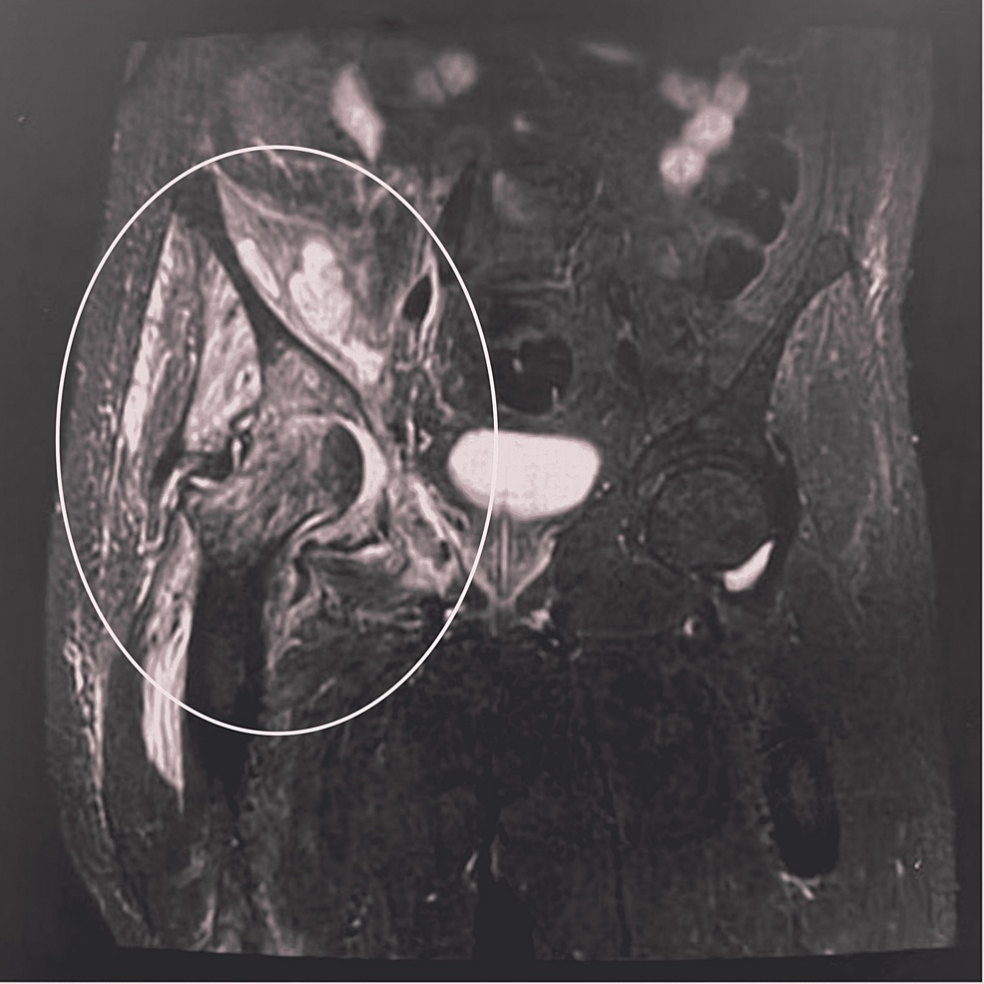
Pelvic MRI showed right hip septic arthritis and large iliacus muscle abscess.

The patient was urgently admitted jointly under Orthopedic and Medicine for magnetic resonance imaging (MRI) and further investigations. An urgent MRI in the early morning showed right hip septic arthritis and osteomyelitis in the surrounding bones with a large abscess in the iliacus muscle (Figure 2).

**Figure 3:**
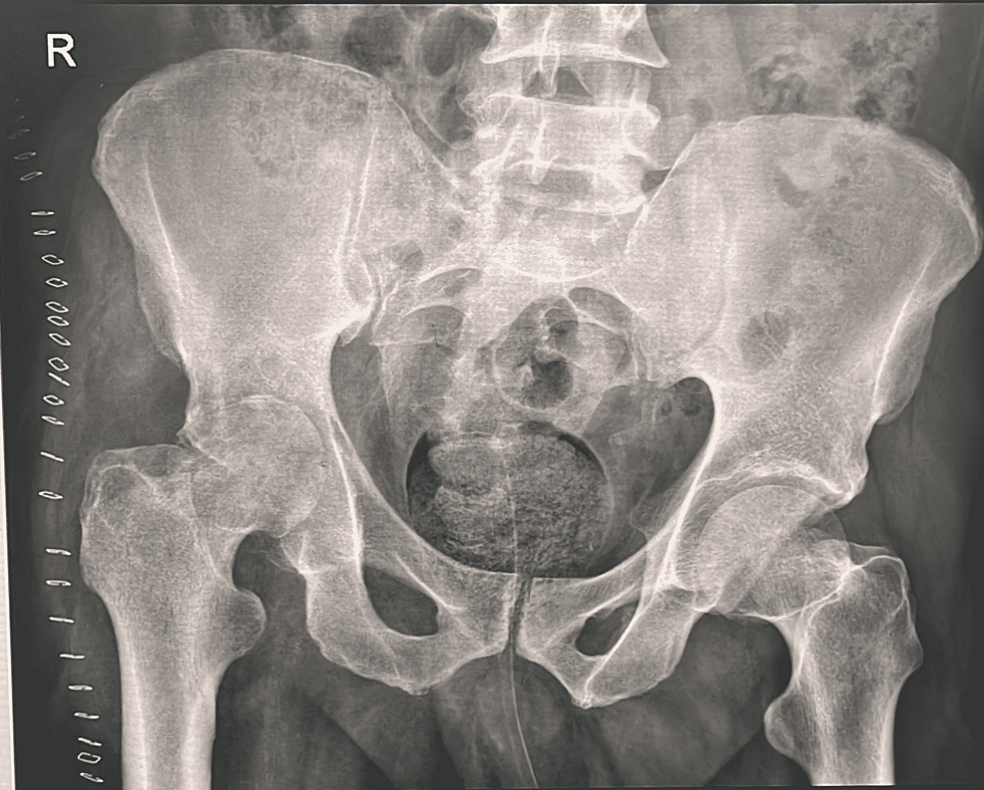
Pelvic x-ray showed severe right hip arthritic changes.

We prepared him for emergency irrigation and debridement after stopping the empirical antibiotic piperacillin/tazobactam 4.5 g I.V every six hours and vancomycin 1250 mg I.V every twelve hours for proper fluid and soft tissue culture and sensitivity. The patient consented to the procedure after knowing the pros and cons. Then the patient was shifted to the operating room and under spinal anesthesia, the anterior approach to the right hip was carried out in a supine position, approaching the iliac crest proximally and the hip joint distally. There was a massive effusion inside the hip joint and massive pus collection around the insertion of the iliopsoas muscle; sample was obtained for culture, sensitivity, and histopathology, and irrigation and debridement of all non-viable tissue was achieved with three drains inserted. X-ray post-debridement showed severe osteoarthritic changes (Figure 3).

**Figure 4:**
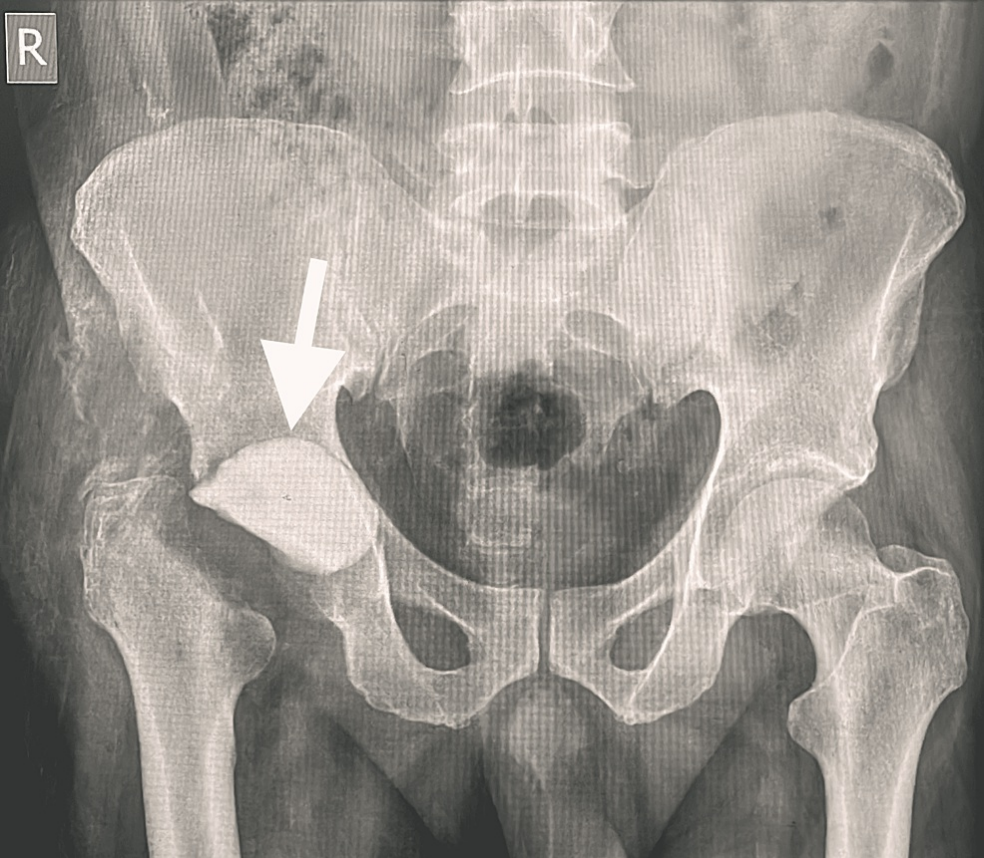
Acetabular cavity cement spacer with antibiotic.

The Infection department recommended cefazoline 1000 mg IV every eight hours as a result of tissue culture results, which showed growth of isolated organism MRSA. Drains were removed on the ninth postoperative day. Repeat laboratory investigations showed elevated WBC count of 12.5 x10^9/L, ESR 55 mm/hr, and CRP 99 mg/L. The patient consented for repeat irrigation and debridement that extended medially and proximally. We found pus turbid and mixed with blood, the acetabulum bone was eroded with soft tissue, and fibrous membrane covering the acetabular bone. There was no cartilage or sclerotic bone; the femoral head and acetabular bone were soft and deformed. The entire femoral head was, thus, excised along with the femoral neck, aspirated fluid, and tissue. Bone samples were sent for histopathology, culture, and sensitivity. Two cement packs mixed with 2 g gentamicin and 6 g vancomycin was placed into the acetabular cavity as cement spacer (Figure 4) along with local antibiotics.

**Figure 5:**
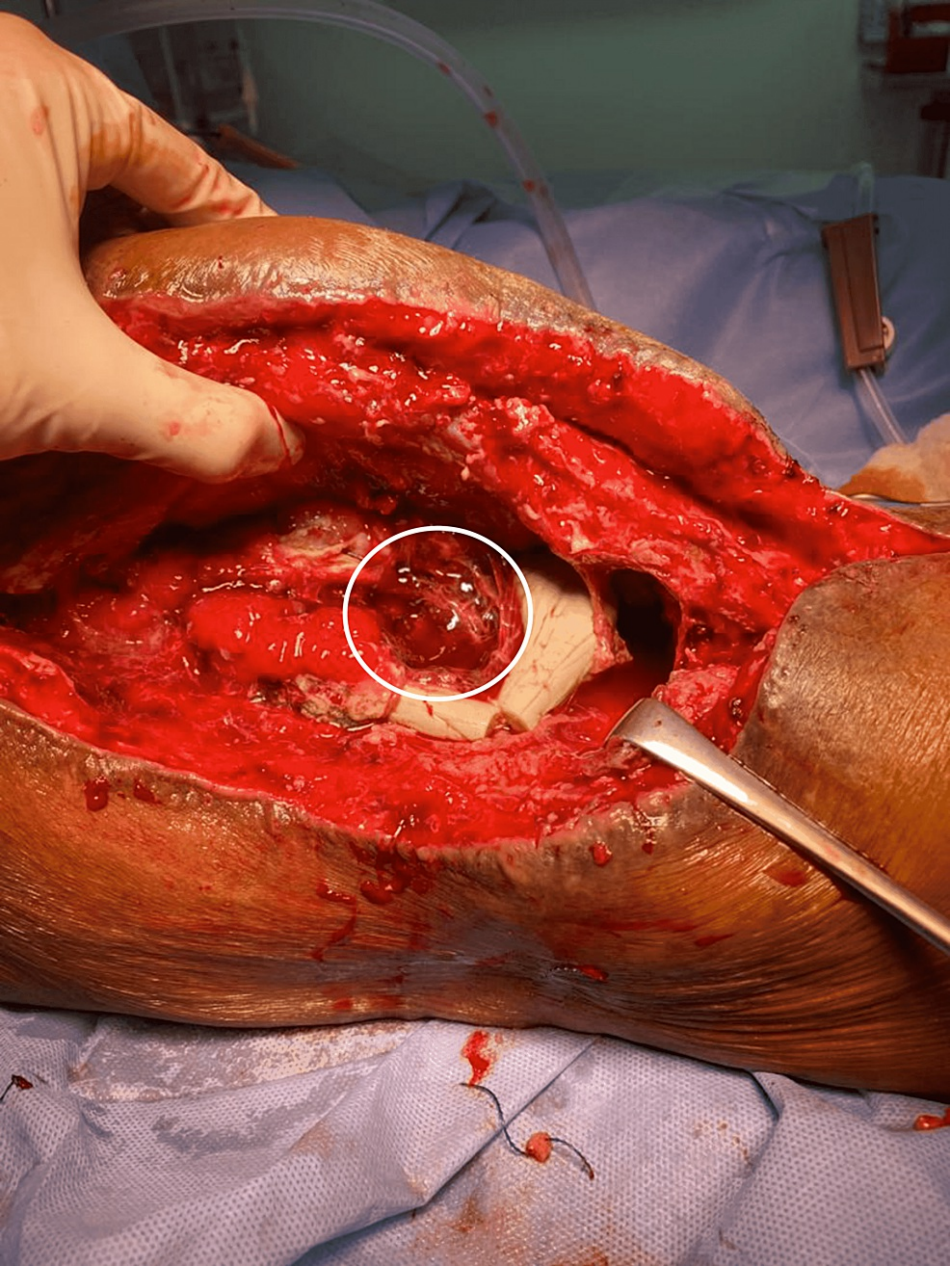
Infected cement spacer: irrigation, debridement, and cement spacer replacement.

Two drains were placed into the hip cavity and a closure of the wound was done layer by layer. It was then covered by a sterile dressing, and the patient shifted after to the recovery area safely. After 35 days from the last surgery on cefazoline 1000 mg IV every eight hours, the patient started to have high inflammatory markers. His ESR increased from 15mm/hr to 52mm/hr and CRP increased from 99mg/L to 157mg/L. The patient was prepared for the third time for irrigation, debridement, and replacement of the infected cement spacer. The aspirated fluid and tissue were sent for for culture and sensitivity (Figure 5).

**Figure 6:**
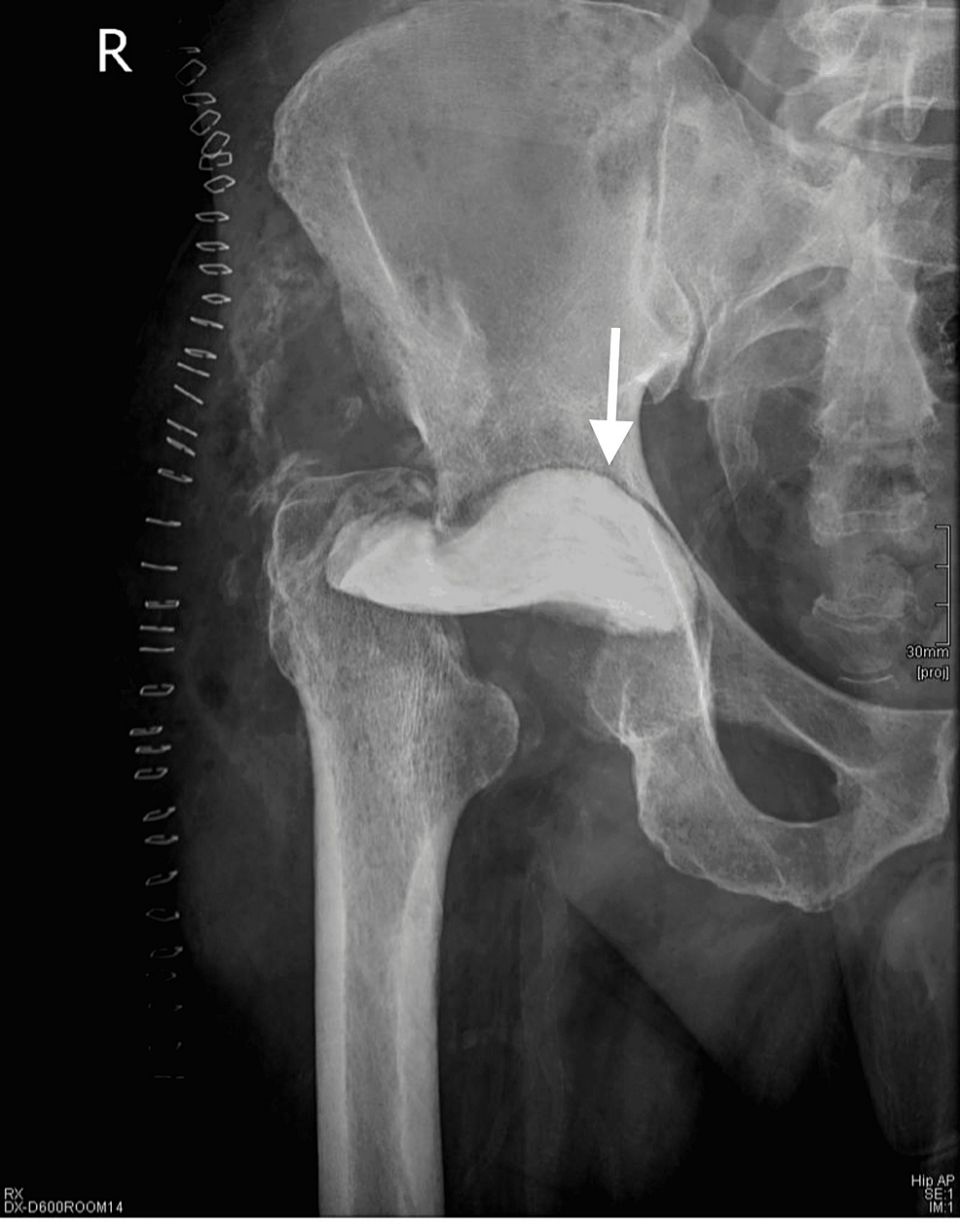
Right hip x-ray after the insertion of the second antibiotic cement spacer.

Utilizing the previous Smith Peterson approach, we obtained fluid aspiration and tissue samples for culture and sensitivity from different areas. The cement spacer was replaced by a new cement spacer composed of 2 g gentamicin mixed with 6 g vancomycin (Figure 6).

The result of tissue culture showed *Pseudomonas aeruginosa* and we started ceftazidime 2000mg IV every eight hours according to Infection department. Laboratory investigations were done twice weekly to see the improvement. After 42 days on ceftazidime, we started to get average levels of the inflammatory markers, ESR 20 mm/hr, CRP 3.5 mg/L, and WBC 4 x10^9/L. Thus, we discharged him on oral antibiotic ciprofloxacin 500 mg every 12 hours for 28 days and weekly follow-up in the clinic for wound examination along with dressing and lab investigations to make sure there is no recurrent hip infection and sufficient treatment plan.

**Figure 7:**
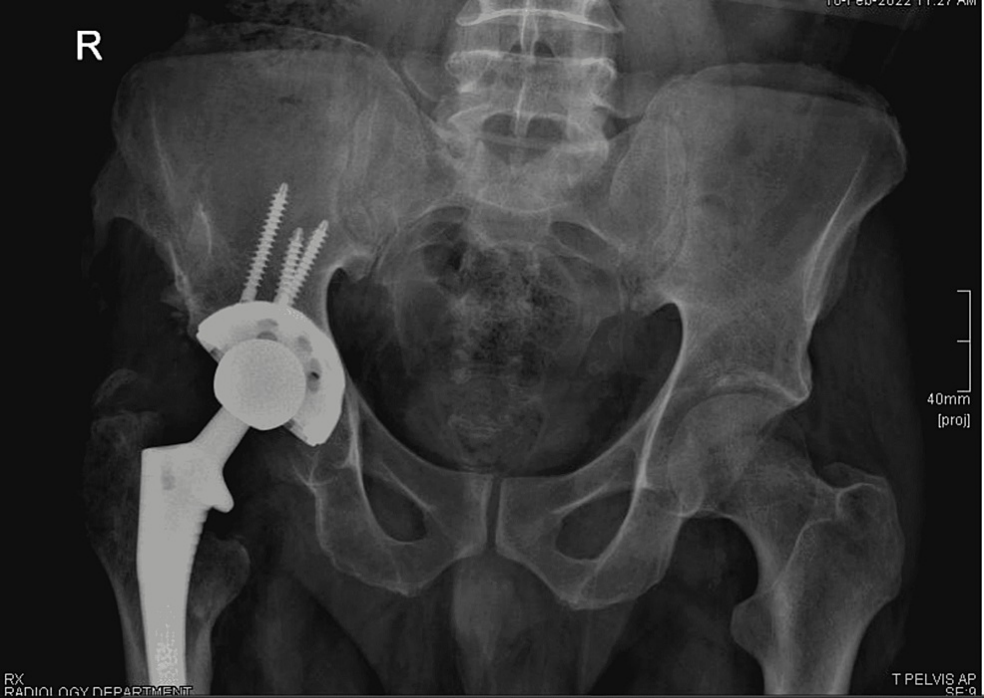
Pelvic x-ray after total hip replacement.

After confirming that there was no remaining infection in the hip joint by fluid aspiration and inflammatory markers, the patient was admitted for cement spacer removal and total hip arthroplasty was done after seven months of the third debridement through lateral approach (Figure 7). 

Our patient started mobilization out of bed using a walking frame from postoperative day one and was discharged home on postoperative day 10, after being fully mobilized. We kept following the patient in the clinic for the next six months after the operation. The surgical wound healed without any discharge, the right hip had full range of motion, and he was ambulating well without any support. At his six-month follow-up, lab investigations showed WBC 6.6 x10^9/L, ESR 33 mm/hr, and CRP 9 mg/L.

## Discussion

IPA was first described as psoitis by Mynter in 1881 [3]. IPA is categorized into primary or secondary causes depending on the etiology behind it. In addition, these abscesses rarely cause septic arthritis and more rarely when the causative agent is known to be MRSA. In the literature, we were able to identify only three cases of psoas abscess complicated by septic arthritis due to MRSA infection (Table 1) [4-6].

**Table 1:** A brief review of the literature on MRSA-positive psoas abscess complicated by septic arthritis.

Authors, year	Type of study	Number of cases	Age, sex of patient	Causative agent	Presentation	Treatment, follow-up
Ash et al., 1995 [4]	Case report	1	57, M	MRSA	Acute presentation requiring immediate intervention	Antibiotics, surgical drainage, and total hip replacement, patient was well and resuming activities after one year.
Ogasawara et al., 2015 [6]	Case report	1	50, F	MRSA	Gradual onset	Abscess drainage under CT with antibiotics.
Al-Zaim et al., 2014 [5]	Case report	1	neonate	MRSA	Gradual onset	Antibiotics and Surgical drainage, patient was well after 13-month follow-up and more assessment was needed for hip function.
Current case report	Case report	1	53, M	MRSA	Gradual onset	Antibiotics, Surgical drainage, and total hip replacement, patient was well and resuming activities after six months.

Primary IPA is thought to be caused mostly due to hematogenous spread of infection [7]. The most common cause of primary IPA has been demonstrated to be *Staphylococcus aureus* conveying 88% in Ricci et al.'s study and, usually, these patients who are affected by primary IPA are immunocompromised [7,2]. Nevertheless, the patient in this report did not have any co-morbidities and was infected by MRSA, which is rarely seen. The suspicion of such pathology is not high in such patients since primary IPA mostly affects those who have an underlying issue such as diabetes or AIDS.

Patients who present with IPA do not have a specific complaint; therefore, the presentation is considered ambiguous [7]. Our patient only complained of hip pain and was diagnosed after one month after his first presentation. Moreover, although the presentation of this pathology is unclear and may appear gradually, early diagnosis is important since the involvement of the hip joint may cause severe deformities, as in our case, or high mortality as a consequence of bacteremia [7]. Hence, the threshold should be raised when dealing with patients who present with long-standing hip pain in the view of normal hip radiographs, and a CT scan with IV contrast, which is the method of choice for diagnosing IPA, should be obtained [8].

The treatment of IPA should include both antibiotics and drainage of infective material. The drainage can be done either surgically or guided by CT [9]. The treatment should be started promptly as delay will increase mortality [2]. Since there was an involvement of the hip joint in our patient, irrigation and debridement was carried out along with drain insertion and appropriate antibiotics.

## Conclusions

IPA can carry a possible risk of septic hip arthritis and permanent damage to the hip joint. Our case highlights the importance of vigilance in anticipation of such a disease and the sequelae of its late diagnosis. At the same time, it can be prevented by a high level of suspicion and proper investigations.
